# Overview of Folic Acid Supplementation Alone or in Combination with Vitamin B12 in Dairy Cattle during Periparturient Period

**DOI:** 10.3390/metabo10060263

**Published:** 2020-06-25

**Authors:** Muhammad Zahoor Khan, Adnan Khan, Jianxin Xiao, Jinhuan Dou, Lei Liu, Ying Yu

**Affiliations:** 1 Key Laboratory of Animal Genetics, Breeding, and Reproduction, Ministry of Agriculture & National Engineering Laboratory for Animal Breeding, College of Animal Science and Technology, China Agricultural University, Beijing 100193, China; zahoorkhattak91@163.com (M.Z.K.); dr.adnan93@cau.edu.cn (A.K.); doujinhuan_cau@163.com (J.D.); liulei03@caas.cn (L.L.); 2 State Key Laboratory of Animal Nutrition, Beijing Engineering Technology Research, Center of Raw Milk Quality and Safety Control, College of Animal Science and Technology, China Agricultural University, Beijing 100193, China; dairyxiao@gmail.com; 3 Genome Analysis Laboratory of the Ministry of Agriculture, Agricultural Genomics Institute at Shenzhen, Chinese Academy of Agricultural Sciences, Shenzhen 518120, China

**Keywords:** folic acid, vitamin B12, dairy cattle, periparturient period, metabolism, milk production, immunity

## Abstract

The periparturient period is the period from three weeks before calving to three weeks post-calving. This period is important in terms of health, productivity and profitability, and is fundamental to successful lactation. During this period, the animal experiences stress because of hormonal changes due to pregnancy and the significant rise in milk production. In addition, a negative energy balance usually occurs, because the demand for nutrients to sustain milk production increases by more than the nutrient supply during the periparturient period. The immunity of dairy cattle is suppressed around parturition, which increases their susceptibility to infections. Special care regarding nutrition can reduce the risks of metabolism and immunity depression, which dairy cattle face during the periparturient span. Folic acid is relevant in this regard because of its critical role in the metabolism to maintain lactational performance and to improve health. Being a donor of one-carbon units, folic acid has a vital role in DNA and RNA biosynthesis. Generally, the folic acid requirements of dairy cattle can be met by the microbial synthesis in the rumen; however, in special cases, such as during the periparturient period, the requirement for this vitamin strictly increases. Vitamin B12 also has a critical role in the metabolism as a coenzyme of the enzyme methionine synthase for the transfer of a methyl group from folic acid to homocysteine for the regeneration of methionine. In the current review, we highlight the issues facing periparturient dairy cattle, and relevant knowledge and practices, and point out future research directions for utilization of the associated vitamins in ruminants, especially during the periparturient period.

## 1. Introduction

The periparturient period is the time span from 3 weeks prepartum until 3 weeks postpartum [1], and is crucial for the reproductive and productive competency of dairy cattle [2]. The peripartum period is characterized by a negative energy balance, immune suppression, and increased susceptibility to diseases in dairy cows [3]. The negative energy balance in periparturient cattle mainly develops due to a rapid increase in milk production during early lactation and dry matter intake depression [4,5]. Pregnancy burden is also one of the reasons for the low feed intake around parturition, resulting in low energy levels. Although the feed intake increases soon after calving, due to the high demand for milk production, the animal continues to be affected by a shortage of energy, which also leads to a negative energy balance [6,7]. Periparturient dairy cattle experience an alteration in the hormonal, digestive, and immune system, which intervenes in the immune function and causes immunity suppression [8,9,10]. Due to immunity depression, the periparturient period is considered an infection-prone period for dairy cattle [11]. 

### 1.1. Immunity and Periparturient Period

During the periparturient period, immune depression in dairy cows is mainly aggravated by physiological changes and negative energy balance [12,13,14]. It is well established that increases in the cortisol level near parturition results in humoral and cell-mediated immunity depression [15]. Many studies have reported the elevated level of non-esterified fatty acids (NEFA) and beta-hydroxybutyrate (BHBA) during the periparturient period, which causes depression of bovine peripheral blood mononuclear cells (PBMCs) [16], inhibits the interferon-γ production [17], and impairs the function of polymorphonuclear neutrophils (PMNLs) [18]. Additionally, the bactericidal efficiency of phagocyte and blood polymorphonuclear leukocytes (PMNs) is severely decreased during the first week of lactation [19]. This decrease in PMNL level exposes dairy cattle to mammary and uterine infections [20]. The process of parturition alters the gene expression profile in neutrophils, which influence the immune system of dairy cattle [21]. Moreover, during the transition period in dairy cattle, the cells involved in the phagocytosis and bacteria encounter activity [18], and immunoglobulin production by B-cells are affected [22]. Studies have shown that the occurrence of metabolic and production-related diseases, such as mastitis, metritis, milk fever, and ketosis, rises in dairy cattle during the periparturient period [23,24].

To cope with this situation, proper nutrition is essential for the normal performance of dairy cows, particularly during the periparturient period [25], because nutrition works as a backbone for the maintenance of good health [26]. Many food supplements have been provided during the periparturient period to avoid stressors, such as immunity depression, negative energy balance, and low dry matter intake due to pregnancy, and to maintain the normal metabolism and productive efficiency of dairy cows. In this regard, folic acid, alone or in combination with vitamin B12, has received considerable attention in ruminant research.

### 1.2. Folic Acid and Vitamin B12 Functions

**Figure 1:**
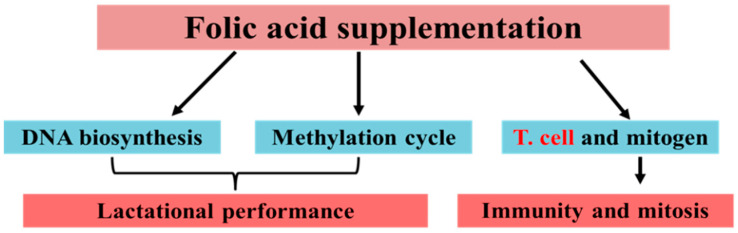
Folic acid regulates the remethylation cycle of methionine, DNA biosynthesis, and T cells.

Folic acid is a B-complex vitamin for which the primary biochemical function in animals is to provide the one-carbon unit [27]. Folic acid has multiple roles and has received considerable attention in animal studies, particularly during pregnancy. Folic acid has multiple functions, including neurotransmission regulation and gene expression [28,29], and has a protective role in the immune system [30,31], while its deficiency leads to anemia, granulocytopenia, and lymphocytopenia [32,33,34]. Hollingsworth reported that the utero exposure to the methyl donor might affect the expression of the critical gene that plays a central role in immunity [35]. In addition, folic acid in combination with B12 improves the efficiency of the energy metabolism in dairy cattle [36,37]. Vitamin B12 works as an essential coenzyme for the methionine synthase, which is necessary for the transfer of a methyl group from 5-methyl-tetrahydrofolate to homocysteine for the regeneration of methionine. The methionine, in the next step, changes to a major methyl group donor called S-adenosyl-methionine (SAM) [38]. Thus, the deficiency of B12 may lead to a reduction in methionine supply and de novo synthesis of methyl groups. It has been reported that a deficiency of folic acid can lead to a decrease in the levels of SAM [39,40]. Folic acid is also involved in T cell and mitogen regulation, which is essential for immunity and growth. Moreover, folic acid influences the methylation cycle, and DNA and RNA biosynthesis, which is essential for lactational performance (Figure 1). In addition, dairy cows have a high demand for methyl groups during early lactation [41]. Due to its central role in accepting and releasing one-carbon units in mammals, folic acid is essential for the de novo synthesis of methyl groups for the formation of methylated molecules [42]. Thus, the adequate supply of folic acid is highly desirable for optimal metabolic pathways and milk production during the periparturient period [43].

Changes in folic acid can alter the activity of methylenetetrahydrofolate reductase (*MTHFR)* and methionine synthase reductase (*MTRR*) genes, which have an essential role in chromosomal stability. Although the bacteria in the rumen can synthesize folic acid [44], under special conditions, such as during the periparturient period, the folate synthesized in the rumen by bacteria is not sufficient to fulfill the requirements of dairy cows. Furthermore, it was noted that folic acid transfers to the fetus in the uterus after absorption from the small intestine of pig [45], and the storing capacity of folic acid increases in the fetus during the late gestation period. The serum folate decreases by up to 40%, two months before calving in dairy cattle [46]. Similarly, a low concentration of B12 was also noted in dairy cattle during early lactation [47]. Thus, the current review aims to summarize the role of folic acid alone or in combination with vitamin B12 to regulate the metabolism, milk production, and immune system in periparturient dairy cattle. In addition, the review highlights the limitations and future research directions for folic acid as a supplement to the ruminant’s diet, specifically around parturition. 

## 2. The Research Progress of Folic Acid and Vitamin B12 in Dairy Cattle

### 2.1. Folic Acid Significantly Regulates Immunity in Periparturient Dairy Cattle

**Figure 2:**
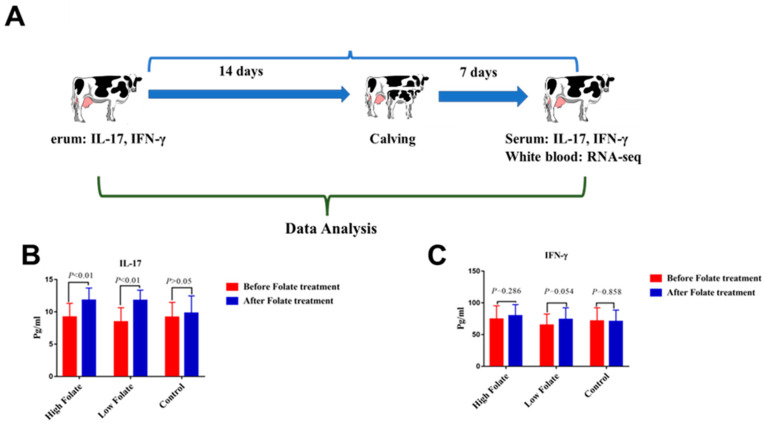
The influence of folic acid supplementation on serum cytokines of periparturient dairy cows [49]. (**A**) Experimental procedures of the entire study. (**B**) The level of IL-17 and (**C**) IFN-γ (pg/mL) in Holstein dairy cattle before and after folic acid supplementation. High folate (240 mg/500 kg), low (120 mg/500 kg body weight).

Interferon-gamma (IFN-γ) is involved in antibacterial activity. It has been noted that, during the periparturient period, the level of IFN-γ is significantly decreased [48]. Recently, our research team observed that folic acid treatment increases the level of IFN-γ and interleukin 17 (IL-17) [49], as shown in Figure 2.

The transcriptomic profiling of peripheral blood lymphocytes in Chinese Holstein showed that folic acid treatment regulated several differentially expressed genes (DEGs), such as *C1QB, ANXA1, CSF1, JSP.1, CCL5, CCL8, CCL16, CXCR5, HCK, MYD88, NFKBIA, MMP9, C1QB, SERPING1, TNFSF13, FOS, NFKBIA, NFKBIE,* and *IL9R,* which have a strong association with immunity and anti-inflammation [49]. In addition, folic-acid-supplementation-mediated *CD4, CD59, C8G, C8B, C8A, FOS,* and *NFKBIA* in periparturient dairy cattle, which are the key genes of the immunity-associated pathways, e.g., T cell signaling, *Staphylococcus aureus* infection, and complement and coagulation cascades [49]. Furthermore, it was found that folic acid supplementation causes the down-regulation of NFKB inhibitor alpha (*NFKBIA*) and TNF alpha-induced protein 3 (*TNFAIP3*), which directed the negative mediation of NF-kappaB transcription factor activity. The suppressor of cytokine signaling 3 (*SOCS3*) was also down-regulated, which is involved in the negative mediation of cytokines that signal through the JAK/STAT pathway. The myeloid differentiation primary response 88 (*MYD88*), nucleotide-binding oligomerization domain containing 2 (*NOD2*), and mitogen-activated protein kinase 13 (*MAPK13*) are the positive mediators of interleukin 6 (IL-6), and were up-regulated by folic acid supplementation in periparturient dairy cattle [49]. The study conducted by Ouattara and colleagues in 2016 attempted to elucidate the effects of folic acid and vitamin B12, given separately or combined in two kinds of tissues (liver and mammary gland) of periparturient cows, to regulate their metabolism and milk production, and establish the molecular mechanism under which these changes take place. Moreover, some important genes, such as *DLK1, LOXL4, SAA3, LOC100126815, MGC126945,* and *IGLL1*, were reported to be mainly engaged in B-cell-mediated immunity, macrophage activation, and the apoptotic process [50]. The distribution of each lymphocyte and granulocyte subset is essential for the immune responses [51], while it has been shown that a folate-deficient diet in rats is associated with a decline in the total number of circulatory lymphocytes and granulocytes [33]. Recently, a study illustrated that folic acid regulates the immunity linked genes (*IL2RG, TLR2, IRF1, IRF7, IRF8, CD40, DQB, RSAD2, ICOSLG, MX1*, and *MX2*) and their pathways (immune response signaling, Toll-like receptor signaling pathway, cytokine–cytokine receptor interaction, and the NF-kappa B signaling pathway) in Hu sheep offspring [52]. From the above-mentioned published studies, it has been concluded that folic acid regulates immunity and could be the best choice to overcome immunity suppression, which is the primary concern in periparturient dairy cattle. Additionally, very limited development of the association of folic acid/vitamin B12 with immunity regulation in periparturient dairy cattle has been undertaken. Thus, it is suggested that trials be extended to provide more in-depth evidence.

### 2.2. Folic Acid Regulates Mastitis Resistance and Down-Regulates the Genes Involved in Mastitis Development

In previous research, we documented some important immunity and anti-inflammatory-associated genes in response to folic acid treatment [49]. The selected genes from previously published data [49] and recent *Staphylococcus aureus* infected mastitis cattle studies [53,54] are shown in Table 1. 

Genes such as *NFKBIA, SOCS3,* and *PIM1*, which play a key role in the development of mastitis, were noted to be up-regulated in the *S. aureus*-infected mammary gland tissue of dairy cattle [53]. *NFKBIA* and *TNFAIP3* play a key role in the negative mediation of NF-kappaB transcription factor activity. *SOCS3* negatively regulates IL-6 [56], which is the key player in immune regulation and has an important role in mastitis resistance. *SOCS3* is also responsible for the negative regulation of cytokines that signal through the JAK/STAT pathway [57]. These two pathways play an essential role in immunity and anti-inflammation. However, the mentioned genes were significantly down-regulated by folic acid administration in periparturient dairy cows [49], as shown in Table 1. Huang and his team concluded that maintaining a low expression of *SOCS3* is also needed for milk synthesis well [57]. *ARG1* plays a critical position in the identification of bacterial sources, while *PTX3* is helpful in the apoptosis process. Additionally, our research team documented some essential signaling, such as antigen presentation, processing signaling, *Staphylococcus aureus* infection, and Toll-like receptor pathways, in response to folic acid supplementation in dairy cattle [49], which were potentially associated with mastitis resistance [53,54]. Moreover, the down-regulation of *KIT* and *LPL* genes negatively affects immunity and also interferes in the metabolic process [53]. In contrast, our research team noted the up-regulation of the mentioned genes [49]. By comparing the gene expression status in the above-published studies (Table 1), we expect that the role of folic acid as a key regulator of many immunity-associated genes might be target for mastitis control research in the future. 

### 2.3. Folic Acid Alone or in Combination with Vitamin B12 Improves Milk Production and Metabolism in Periparturient Dairy Cattle

**Figure 3:**
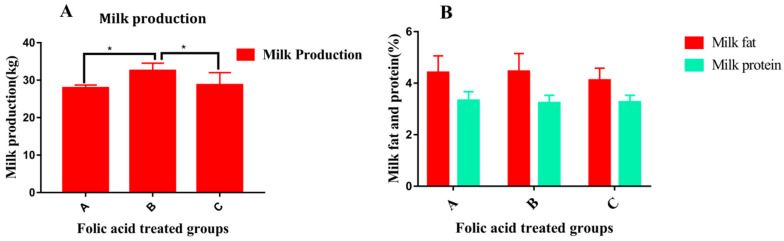
The effect of folic acid quantity on milk production variables in periparturient dairy cows [58]. (**A**) Comparison of the average milk production status over three months in group B (120 mg/500 kg body weight/cow), group A (240 mg/500 kg body weight/cow), and group C (control). (**B**) Comparison of average milk fat and protein percentage in three folic acid-treated dairy cattle groups (A, B, and C). The * denotes that the difference is significant and the *p*-value < 0.05.

Recently, our research team investigated the association of folic acid supplementation alone with milk production and milk variables [58], as shown in Figure 3. Furthermore, it was also observed that the quantity of folic acid plays a role in lactational performance [58].

Note: Overall, Figure 3 shows that treatment in group B was effective for the improvement in milk production. In addition, folic acid treatment in all groups (A, B, C) did not show any effect on milk variables (milk fat and protein).

Folic acid supplements increase milk yield and milk protein in dairy cattle [43,59,60,61,62,63]. However, some studies did not note any effect of folic acid on lactational performance, which might be due to the low concentration of vitamin B12 in the plasma of dairy cattle [64,65]. Folate metabolism seems to have a vital influence on milk protein synthesis in mammary epithelial cells [66]. Recently, our published study noted that oral supplementation of folic acid significantly regulates milk production in periparturient dairy cows [58]. The effects of folic acid on milk production performance may be due to its correlation with DNA synthesis and the methylation cycle. The above studies showed that folic acid plays an essential role in milk yield improvement and should be supplied to periparturient dairy cattle.

A recent study [58] performed transcriptomic profiling of blood lymphocytes of folic acid-treated periparturient dairy cattle. The findings of the analysis illustrated that folic acid treatment mediated key DEGs (*LAP3, SOCS3, BMX, ZC3H12A, UBE2C, MRPL57, BRCA1, IGF1R, LPL, HK3, PKM, VLDLR, XDH, SLC25A25, CTSB, DGAT2, UBE2R2, BRCA1, GGT, CALCRL, SDSL, ALOX5, SPP1, LYZ*, and *PPARD*) that were notably associated with metabolism, milk fat, protein, milk production, and mammary gland development [58]. Cui and colleagues recorded several genes, such as *RPL23A, SLC25A38, BMX*, and *ZC3H14,* with a strong correlation with milk fat and protein yield [67]. Similarly, *DGAT2, UBE2R2, BRCA1, VLDLR*, and *CTSB* genes show an association with milk production [68]. The calcitonin receptor-like receptor (*CALCRL*) serves as a receptor for the further action of calcitonin genes. Using RNA-Seq, Seo and co-workers demonstrated that calcitonin genes are extensively linked to milk fat yield [69]. Two genes, namely, *SPP1* and *LYZ*, were predominantly involved in the growth of mammary tissue and milk yield in Rhesus macaques [70]. Similarly, *GGT, SDSL, PPRAD*, and *ALOX5* regulate pathways associated with mammary gland development and fatty acid metabolism, and are also involved in milk production [71]. 

From the above-mentioned published data, it has been shown that the response of mRNA to folic acid in periparturient dairy cows is close to that observed for the phenotypic milk production outcome. Furthermore, the reported genes associated with milk production in the above studies in response to folic acid might be targets as milk improvement markers in dairy cattle. The high production of milk raises the energy demand, resulting in a negative energy balance occurring in the body [7]. Moreover, the studies regarding folic acid and vitamin B12 treatment during the periparturient period in dairy cattle have revealed that proper metabolism is a crucial requirement to maintain milk production.

**Figure 4:**
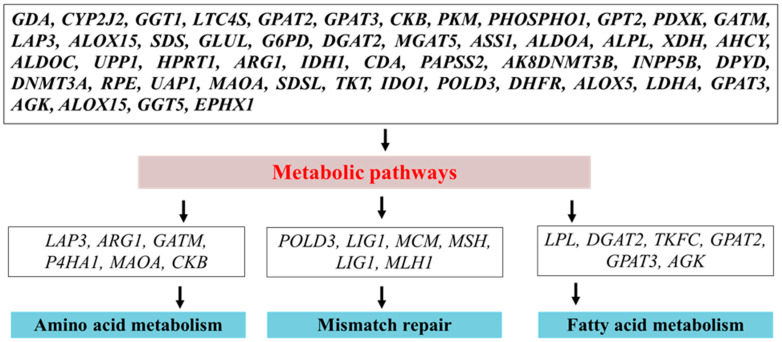
Folic acid supplementation mediates the essential genes linked to metabolic pathways (amino acid and fatty acid) and mismatch repair. For ease, the genes mainly involved in metabolic pathways, mismatch repair, fatty acid metabolism, and protein synthesis are summarized.

Folic acid is essential for the growth of ruminal bacteria and boosts the population of the cellulolytic bacterial population and the digestion of cellulose [72]. Intake of dry matter will normally decline during the peripartum period. However, the rumen-protected treatment of folic acid substantially promotes the intake of dry matter and also promotes the metabolism and energy balance [73]. Although the major absorption site of folic acid is the proximal duodenum, however, some portions of folic acid are also absorbed in the rumen [74]. Folic acid supplementation promotes metabolic effectiveness, dry matter intake, and overall fiber degradability in dairy cattle [72,73]. It was revealed, through a molecular-based mechanism, that folic acid and vitamin B12 regulate several genes that are associated with metabolism and milk yield. Metabolic pathways engage in a chain of chemical reactions that take place in the cell. These metabolic pathways are associated with either synthesizing molecules by utilizing energy or breaking down molecules, or releasing energy in the process known as the catabolic pathway [75]. Pathways are important for sustaining homeostasis within an organism, and the flow of metabolites through a pathway is controlled based on the cell’s needs and the substrate’s availability. We also noted in our previously published study that the majority of genes demonstrated their importance for the mediation of metabolic signaling, which further speeds up various biological processes related to metabolism [58]. Folic acid acts in the acceptance and release of one-carbon units [27], which are vital for the biosynthesis of purine, pyrimidine, and DNA, and also offers the methyl group for the formation of S-adenosylmethionine. Methyl-tetrahydrofolate is the cofactor providing a methyl group for remethylation of homocysteine into methionine, and this reaction is irreversible. Methionine works as a substrate for SAM, a cofactor and methyl group donor for many methylation reactions, such as methylation of DNA, RNA, proteins, and phospholipids [76]. The inadequate supply of folic acid may lead to a DNA mismatch repair and abnormal cell growth because of the essential role in the synthesis of DNA. Similarly, in a recently published study, many essential genes, such as *ALDOA, PKM, SDS, SDSL, ASS1, POLD3, XDH*, and *ARG1*, in response to folic acid treatment, were documented as being significantly associated with the biosynthesis of amino acid signaling [58]. Moreover, *MCM2, MCM3, POLD3*, and *LIG1* were documented to have an involvement in DNA replication and mismatch repair, while *PHOSPHO1, LPCAT2, GPAT2*, and *GPAT3* were found to control the glycerophospholipid metabolism. For milk lactose synthesis in dairy cattle, glucose is essential, and is provided by the processes of glucogenesis and glycogenolysis [77]. The combined supplement of folic acid and vitamin B12 promote gluconeogenesis in periparturient dairy cattle [65], while a recently published study has also shown the association of folic acid linkages to the regulation of glycolysis/gluconeogenesis and related genes, i.e., *LDHA, ALDOC*, and *ALDH3B1* [58]. The metabolic-associated genes further facilitate the biological function processes (Figure 4). 

Additionally, HERC6, PPP1R3B, PYCR1, ID1, MYOM1, CDK5R1, DLK1, HP, LOXL4, FUT5, HERC6, LOC515676 ISG15, PYCR1, and CACNA2D1 were noted to be associated with metabolic processes, while MEP1B, DLK1, MYOM1, SFRP1, MEP1B, IGLL1, and SPP1 genes were linked to developmental functions [58]. The above-mentioned published research revealed that folic acid, in combination with vitamin B12, may be the best option for maximizing the milk production and metabolic efficiency of periparturient dairy cows.

## 3. Future Direction for Research of Folic Acid and Vitamin B12 in Transition Dairy Cattle

Based on the published studies on folic acid and vitamin B12, we note that, although folic acid has a crucial role in disease control, as shown in human and mouse studies, to date, no study has established the association of folic acid with disease prevention in dairy cattle. Thus, we suggest that further research is needed in dairy cattle to examine the influence of folate supplementation, alone or in combination with vitamin B12, on disease control. As our recently published study documented the association of folic acid supplementation with mastitis-linked genes, it would be of interest to extend the study in further mastitis research. To date, the exact requirement of folic acid supplementation has not yet been reported in periparturient dairy cattle research. Thus, it is an important topic for future research, because both low and high levels of folic acid may have serious consequences. In addition, it has been proved that high doses of folic acid are toxic and reduced the natural killer cells (NK) cytotoxicity responsible for the regulation of the innate immunity system in mice [78].

This review showed that controversy remains regarding the influence of folic acid and vitamin B12 on milk production, and the effect of feed intake on the availability of folic acid in dairy cattle. These issues thus need to be addressed in future research. Additionally, the microorganisms in rumens should be characterized for folic acid needs and synthesis during the transition period. In vitro study is also recommended to examine the individual and combined influence on milk production, metabolism, and immunity. Considering folic acid is a methyl donor, we strongly recommend an epigenomic study to investigate the mechanism by which folic acid influences the epigenetic modifications of different beneficial genes responsible for disease resistance, metabolism, and milk production. 

## 4. Conclusions

Based on the cited literature, we concluded that the periparturient period is vital in dairy cattle; special managemental care, including balanced intake of folic acid and vitamin B12, is needed. The review suggests that folic acid and vitamin B12 are essential nutrients and, as such, should be supplemented in dairy cattle during the periparturient period to overcome metabolic and immune stress. Milk production can be improved with the supplementation of folic acid and vitamin B12, which are key concerns for animal scientists. Folic acid quantity should be considered during supplementation because an excessive intake of folic acid suppressed NK-cells. Folic acid supplementation regulates immunity and mastitis-linked genes. Thus, the targeting of folic acid in mastitis control studies in periparturient dairy cattle is recommended. Genes regulated by folic acid treatments might be a useful addition to the marker selection for milk production and metabolic regulation in peripartum cattle.

## Figures and Tables

**Table 1:** Comparison of gene regulation status in folic acid-treated and *S. aureus*-infected mastitis cows.

Gene	*S. aureus* Treatment	Folic Acid-Treated Group [49]
*PIM1, SOCS3, ATP12A, NFKBIA, DUSP4, ZC3H12, ESPNL, TNFAIP3*	Up-regulated [53]	Down-regulated
*CX3CR1, ALOX5, KIT, LPL*	Down-regulated [53]	Up-regulated
*C1QA, C1QB, CCL5, MMP9, VNN, BLA-DQB*	Up-regulated [54]	Up-regulated
*ICAM1, CXCL10*	Up-regulated [54]	Down-regulated
*PPARD, ARG1, PTX3, CD4*	Up-regulated [55]	Up-regulated
